# Molecular mechanisms controlling plant growth during abiotic stress

**DOI:** 10.1093/jxb/ery157

**Published:** 2018-05-19

**Authors:** Ulrike Bechtold, Benjamin Field

**Affiliations:** 1 School of Biological Sciences, University of Essex, Colchester UK; 2 Aix Marseille Univ, CEA, CNRS, UMR7265 BVME, Marseille, France

**Keywords:** Abiotic stress, Arabidopsis, ascorbate, chloroplast proteome, heat shock transcription factorA1b, (p)ppGpp, phytochrome, plant growth, productivity, retrograde signals

**Mechanisms that protect against abiotic stress are essential for plant survival, yet their activation generally comes at the expense of growth and productivity, which is particularly serious for agriculture. Recent developments in molecular genetics have contributed substantially to our understanding of the basis of abiotic stress defense. Progress has also been made towards understanding how plants control the switch between growth and defense, especially with regard to timing and mechanism. This ongoing research is critical for the improvement of crop plants.**

Cell proliferation and growth require nutrients, biosynthetic capacity and energy. Restricting any one of these factors will lead to arrested growth and eventually death. To ensure their survival it is therefore necessary for living organisms to anticipate changes in the environment that might affect their capacity to grow, and then to mount an effective acclimatory response. This is particularly important in plants, which are typically immobile and encounter large fluctuations in temperature, light, humidity and nutrient availability in their natural environment (see Box 1). Environmental stress causes massive agricultural losses (Godfray *et al.*, 2010; Cramer *et al.*, 2011), and improving crop tolerance is a major goal of crop improvement programs. However, tolerance can come with trade-offs; for example, it has long been known that stress-tolerant plants have lower growth rates and productivity (reviewed by Chapin, 1991). Therefore, in addition to understanding the basis of tolerance, it is also important to understand the trade-offs between tolerance and growth/productivity for effective crop improvement.

**Box 1. Plant growth during abiotic stress**

Carbohydrate resources and energy generated by photosynthesis (circular arrows) are allocated to growth and reproduction. Nutrient limitation or abiotic stress exposure can limit growth and also lead to over-excitation of the photosynthetic electron transport chain and the production of potentially damaging ROS. Timely perception of stress leads to the modulation of plant growth and the activation of defense and acclimation pathways that can act within specific plant organs, or across the entire plant. Key players in the control of plant growth during abiotic stress are shown. Chloro, chloroplast; GA, gibberellins; BR, brassinosteroids; SA, salicylic acid; ET, ethylene.

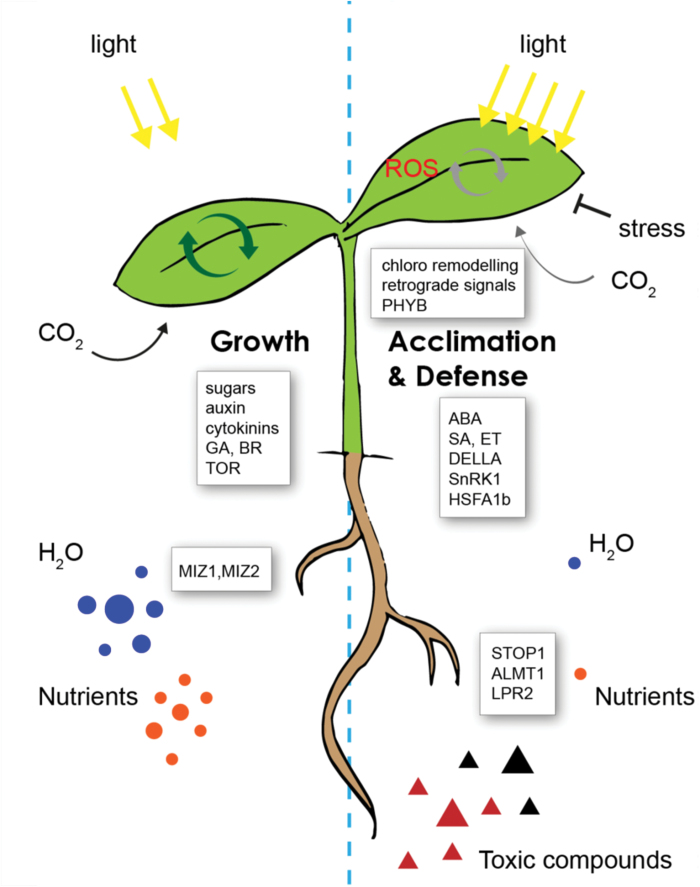

The impact of abiotic stress on plant performance is being explored at many different levels, in a great variety of model and crop species, and includes metabolic/physiological responses, molecular signaling pathways, ecophysiology and crop breeding studies. In addition, abiotic stress is not a single entity but rather comprises all the environmental perturbations that plants may encounter in nature. Consequently, the literature on abiotic stress responses is vast, and covers very diverse research areas. Here, we focus on a selection of recent advances made in our understanding of the molecular mechanisms that control plant growth during abiotic stress.

## Nutrient and water limitation: the root perspective

Nutrient limitation has drastic effects on plant growth and development. Under mild nutrient deprivation plant architecture may be modified to increase nutrient uptake, while severe nutrient limitation may lead to complete growth arrest. Roots are essential for water and nutrient uptake, but also serve a variety of other functions, such as forming symbioses with other microorganisms in the rhizosphere, anchoring the plant to the soil, and acting as storage organs. Consequently, roots are essential for optimal plant productivity. Many abiotic stresses are first encountered at the root level often leading to changes in root biomass and architecture. For example, primary root growth stops when Arabidopsis seedlings are transferred to media without phosphate. This growth arrest is the consequence of a signaling pathway mediated by *STOP1*, *ALMT1* and *LPR2* (Balzergue *et al.*, 2017). Strikingly, knockout mutants of these genes lose the root growth arrest response on phosphate removal, indicating that root growth arrest is not a result of metabolic limitation.

Importantly, when roots encounter changes in environmental conditions they will change growth direction in order to optimize plant survival. Such directional changes in response to stimuli (tropisms) include where roots sense the soil water content and grow towards water to avoid dry soil by either changing direction or halting growth. Despite water sensing being the subject of very early plant physiology studies, until recently the mechanisms of this growth response were essentially unknown. Some genes required for hydrotropism, such as *MIZ1* and *MIZ2/GNOM*, have now been identified (Kobayashi *et al.*, 2007; Miyazawa *et al.*, 2008), and a role identified for the action of plant hormones such as auxin, ABA and cytokinin (Moriwaki *et al.*, 2011; Moriwaki *et al.*, 2012; Saucedo *et al.*, 2012). More recently the site of water perception and growth control was localized to the root cortex (Dietrich *et al.*, 2017), and progress and perspectives in the active hydrotropism field are reviewed in this issue by Dietrich (2018). The review highlights the many outstanding questions that remain regarding the signaling pathways involved in hydrotropism, as well as the need for further research in this area. Indeed, it has been suggested that the genes involved in hydrotropism could be important targets for crop improvement by enhancing drought avoidance. A recent demonstration that a robust hydrotropic response leads to better growth under drought and partial lateral irrigation in different maize cultivars strongly supports this notion (Eapen *et al.*, 2017).

## Growing pains: abiotic stress

Abiotic stress leads to altered biosynthetic capacity and nutrient acquisition that can inhibit plant growth. This phenomenon is documented in many research papers on model and crop species alike. Consequently, research into understanding the responses to abiotic stress has moved to the forefront over the past decade, leading to the discovery of several signaling pathways involving a large number of genes, proteins and post-translational modifications. These include the MAPK, ABF/bZIP, Ca^2+^-CBL-CIPK and CBF/DREB signaling pathways, which employ numerous stress-responsive transcription factors to orchestrate the downstream responses required to mount an effective defense to specific abiotic challenges (Wang *et al.*, 2016; Zhu, 2016).

Importantly, these molecular signaling pathways can anticipate the effects of abiotic stress to regulate the balance between growth and acclimation. More recently, efforts into understanding how plant growth is regulated under stress conditions has resulted in the identification of candidate genes that may integrate both processes. For example, the molecular mechanisms that control leaf growth under mild drought conditions link both growth and transcriptional responses to the circadian clock. Specifically, two *ETHYLENE RESPONSE FACTORS* (ERFs), *ERF2* and *ERF8*, were found to affect leaf growth under drought and well-watered conditions (Dubois *et al.*, 2017). Interestingly, in the same study the specific up-regulation of three genes encoding growth-repressing DELLA proteins was observed during the early drought response (Dubois *et al.*, 2017). DELLA proteins have previously been shown to accumulate under nutrient deficiency, low temperature treatment and in response to salt stress (Achard *et al.*, 2008; Xie *et al.*, 2016). DELLAs promote stress-inducible anthocyanin biosynthesis through the formation of a JAZ–DELLA–MYBL2 complex (Xie *et al.*, 2016) and can also promote ROS scavenging to delay cell death (Achard *et al.*, 2008). Stress-induced anthocyanin accumulation is significantly inhibited in *della* mutants (Xie *et al.*, 2016), while under salt stress *della* quadruple mutants produce significantly more ROS than the wild type (Achard *et al.*, 2008). DELLA proteins therefore promote survival under abiotic stress conditions. Interestingly, reduced anthocyanin accumulation in response to high light was also observed in the ascorbate-deficient mutants *vtc2-1* and *vtc2*-4, yet both *vtc* mutants experienced identical levels of photodamage compared to wild type. This suggests that ascorbate is not essential for photoprotection during high light, but intriguingly is required for the accumulation of rosette biomass under low-light and short-day conditions (Plumb *et al.*, 2018).

Signal transduction pathways mediated by phytohormones can play a critical role in abiotic stress responses (reviewed by Verma *et al.*, 2016). For example, ABA plays a key role in stress responses, while auxin plays a major role in promoting plant growth. The interplay between phytohormones is therefore an important mechanism for balancing growth and stress resistance. Brassinosteroids are a class of plant steroid hormones that promote growth via the activation of the transcription factors BZR1 and BES1. A recent study has shown that drought stress represses the brassinosteroid signaling pathway, and thereby growth, by promoting the degradation of BES1 via ubiquitination and selective autophagy (Nolan *et al.*, 2017). This example highlights the importance that plant hormones can have as major integrators of environmental stress and nutrient status.

## Hunger games: nutrient and energy signaling

Over recent years it has become clear that plants integrate energy/nutrient status to regulate growth and stress responses using antagonistic signaling pathways mediated by the evolutionarily conserved protein kinases TOR (TARGET OF RAPAMYCIN) and SnRK1 (Snf1-RELATED PROTEIN KINASE1) (Robaglia *et al.*, 2012; Broeckx *et al.*, 2016; Baena-González and Hanson, 2017). The central role of these kinases in energy metabolism is underlined by their wide conservation in the eukaryotes, from yeast and animals to plants and fungi (Roustan *et al.*, 2016). SnRK1 is activated by low-energy conditions, such as those that may occur during stress exposure, to trigger catabolism and repress growth. Notably, SnRK1 can be activated by the inhibition of photosynthesis with the inhibitor DCMU, and can be inhibited by the addition of sugars. SnRK1 directly targets metabolic and regulatory enzymes in the cytosol, and also affects gene expression via the phosphorylation of transcription factors such as BZIP63 (Mair *et al.*, 2015; Nukarinen *et al.*, 2016). In contrast, TOR promotes cell growth and proliferation in response to light, sugars, and growth-promoting hormones through the phosphorylation of target proteins (recently reviewed by Schepetilnikov and Ryabova, 2018). Over the past 10 years a growing number of TORC client proteins and downstream effectors have been firmly identified in plants, including the S6 kinase, E2F, and the brassinosteroid pathway. A very recent study has shown that TOR can also phosphorylate the ABA receptor PYL to prevent activation of the ABA-signaling effector kinase SnRK2 in non-stressed plants (Wang *et al.*, 2018). In turn, under stress conditions, ABA is able to activate SnRK2, which then phosphorylates a member of the TOR complex RAPTOR, which triggers complex dissociation and TOR inactivation. This antagonistic signaling loop is an excellent example of how plants are able make the decision between growth and stress acclimation. Interestingly, both TOR and SnRK1 have been implicated in the regulation of chloroplast function (Dong *et al.*, 2015; Dobrenel *et al.*, 2016; Nukarinen *et al.*, 2016; Sun *et al.*, 2016; Imamura *et al.*, 2018).

## It all comes down to light: chloroplasts at the centre of stress perception and regulation

Chloroplasts are one of the powerhouses for plant productivity, but photosynthesis is highly sensitive to light, CO_2_ levels, and plant metabolic capacity. Excess light, or limitation in CO_2_ supply or metabolic capacity, during abiotic stress exposure rapidly leads to over-excitation and reduction of the photosynthetic electron transport chain. Over-excitation is potentially highly dangerous for the plant because it can lead to the production of ROS such as ^1^O_2_ and H_2_O_2_ that can irreversibly damage proteins, membranes and DNA. However, changes in chloroplast redox status during overexcitation act as a signal that leads to the rapid activation of energy-dissipating mechanisms, changes in chloroplast genome expression, and over the longer term to changes in chloroplast protein composition and position to allow acclimation. Importantly, chloroplast stress triggers acclimation at the cellular level as well as the organellar level, and as the severity of stress increases can lead to growth inhibition and eventually programmed cell death (Laloi and Havaux, 2015). The majority of chloroplast proteins are encoded in the nuclear genome. Remodelling of the chloroplast proteome during abiotic stress acclimation therefore requires signaling from the nucleus to the chloroplast (anterograde signaling), and from the chloroplast to the nucleus (retrograde signaling). An overview of chloroplast proteome remodelling, with a focus on stress-regulated import of proteins, nuclear control of the chloroplast genome and protein turnover within the chloroplast is reviewed in this special issue (Watson *et al.*, 2018). Stress-induced retrograde signaling from the chloroplast is also considered from a different perspective by Crawford *et al.* (2018). In particular, these authors discuss how the stress-induced down-regulation of photosynthesis and respiration in the mitochondria can lead to a reduction in the supply of energy available for cellular stress acclimation. They propose a new hypothesis for the integration of different organellar retrograde signals in the nucleus to coordinate transcriptional responses that regulate the allocation of energy to either growth or stress acclimation. Notably, and in relation to this hypothesis, recent work indicates that chloroplast-generated H_2_O_2_ acts as a retrograde signal that is directly transferred from the chloroplast to the nucleus, avoiding the cytosol, to drive a transcriptional response (Exposito-Rodriguez *et al.*, 2017). Stress can also lead to transcriptional reprogramming within the chloroplast, and the signaling nucleotides guanosine tetra- and penta-phosphate [or (p)ppGpp] potentially play a major role (Field, 2018). Indeed, (p)ppGpp is known to accumulate in response to a wide range of different abiotic stresses, and both *in vitro* and *in vivo* studies show that (p)ppGpp accumulation inhibits chloroplast transcription and affects chloroplast function. These findings and other recent advances in our understanding of (p)ppGpp metabolism in plants and algae are reviewed by Field (2018).

While light plays an obvious role in the production of photosynthates and energy, a perhaps less intuitive role is in the regulation of biomass partitioning and plant architecture in response to resource availability, which can occur in a phytochrome B (PHYB) dependent manner (Arsovski *et al.*, 2018). The function of phytochromes as regulators of carbon supply, metabolic status and biomass production has been recently proposed (Yang *et al.*, 2016), and together with the PHYB- and light-dependent development of stomata (Casson and Hetherington, 2014) emphasizes the close connection between light perception and photosynthetic metabolism beyond photosynthetic electron transport. PHYB was also recently shown to act as a temperature sensor in plants. PHYB activity decreases with increasing temperature in a light-dependent manner (Legris *et al.*, 2016), to allow the optimization of growth and biomass production under different environmental conditions. Furthermore, PHYB has been demonstrated to uncouple growth and defense pathways through the relief of transcriptional repression, thereby providing a direct link between light, plant growth and defense signaling pathways (Campos *et al.*, 2016; Cerrudo *et al.*, 2017).

## The trade-off between growth and defense: a balancing act?

In light of the diverse molecular mechanisms that regulate growth and abiotic stress acclimation the question arises as to whether the induction of stress tolerance always leads to growth penalties, or whether we can get something for nothing. It is commonly thought that constitutive stress tolerance comes at a cost to the organism, and this has been extensively reviewed for disease resistance traits (Heil, 2014; Heil and Baldwin, 2002). Early examples of engineered constitutive abiotic stress tolerances have often led to growth penalties under benign growth conditions (Kasuga *et al.*, 1999; Haake *et al.*, 2002). Another example is the *Physcomitrella patens ppabi1a/b* double mutant, where ABA signaling is constitutively active, which is stress resistant but also shows very severe growth defects (Komatsu *et al.*, 2013). However, there are now many indications that the cost need not always be so high. C24, an Arabidopsis ecotype from the Iberian peninsula, is resistant to ROS, heat and drought stress yet shows similar productivity to less-tolerant ecotypes. These features have led to research into the genetic and molecular basis of the growth/resistance equilibrium in C24, and is reviewed in this issue by Bechtold *et al.* (2018). The hope is that research in such a tractable model species may lead to the rapid development of new strategies for conferring stress resistance to crop plants without penalties. The basis of C24 stress resistance is likely to be complex and multigenic. However, even the overexpression of a single transcription factor gene, such as Heat Shock Transcription FactorA1b, can lead to penalty-less increases in abiotic stress resistance (Bechtold *et al.*, 2013), and other positive examples utilizing single-gene manipulations are highlighted in Bechtold *et al.* (2018). Intriguingly, the molecular basis of HSFA1b stress resistance appears to be in its ability to regulate the expression of a large hierarchical network of stress and development genes (Albihlal *et al.*, 2018), suggesting the HSFA1b could be a master regulator of the switch between growth and abiotic stress defenses. It will also be fascinating to discover how such ‘penalty-less’ improvements in stress tolerance are able to bypass SnRK1/TOR-mediated growth control.

## Future directions

Research into plant responses to environmental stress and the application of this knowledge to improve productivity under non-optimal growing conditions is becoming ever more important. Over recent years dramatic progress has been made, and the molecular mechanisms for many stress response pathways revealed. Identification of the cellular hubs that integrate these diverse stress acclimation mechanisms, and the regulatory logic behind the plant’s decision-making processes, are now emerging themes in the field. Over coming years further research in these directions has the potential to lead to a more unified view of plant growth and abiotic stress resistance that could be applied for the rational improvement of crop plants.
